# DNA damage as a mechanism of neurodegeneration in ALS and a contributor to astrocyte toxicity

**DOI:** 10.1007/s00018-021-03872-0

**Published:** 2021-06-26

**Authors:** Jannigje Rachel Kok, Nelma M. Palminha, Cleide Dos Santos Souza, Sherif F. El-Khamisy, Laura Ferraiuolo

**Affiliations:** 1 University of Sheffield, Sheffield Institute for Translational Neuroscience (SITraN), Sheffield, UK; 2 Department of Molecular Biology and Biotechnology, The Healthy Lifespan Institute, Sheffield, UK; 3 The Institute of Neuroscience, University of Sheffield, Sheffield, UK; 4 The Institute of Cancer Therapeutics, West Yorkshire, UK

**Keywords:** Amyotrophic lateral sclerosis, Neurodegeneration, DNA damage, DNA damage response, Astrocytes

## Abstract

Increasing evidence supports the involvement of DNA damage in several neurodegenerative diseases, including amyotrophic lateral sclerosis (ALS). Elevated levels of DNA damage are consistently observed in both sporadic and familial forms of ALS and may also play a role in Western Pacific ALS, which is thought to have an environmental cause. The cause of DNA damage in ALS remains unclear but likely differs between genetic subgroups. Repeat expansion in the *C9ORF72* gene is the most common genetic cause of familial ALS and responsible for about 10% of sporadic cases. These genetic mutations are known to cause R-loops, thus increasing genomic instability and DNA damage, and generate dipeptide repeat proteins, which have been shown to lead to DNA damage and impairment of the DNA damage response. Similarly, several genes associated with ALS including *TARDBP*, *FUS*, *NEK1*, *SQSTM1* and *SETX* are known to play a role in DNA repair and the DNA damage response, and thus may contribute to neuronal death via these pathways. Another consistent feature present in both sporadic and familial ALS is the ability of astrocytes to induce motor neuron death, although the factors causing this toxicity remain largely unknown. In this review, we summarise the evidence for DNA damage playing a causative or secondary role in the pathogenesis of ALS as well as discuss the possible mechanisms involved in different genetic subtypes with particular focus on the role of astrocytes initiating or perpetuating DNA damage in neurons.

## Introduction

### ALS and DNA damage

Amyotrophic lateral sclerosis (ALS) was first described by Jean-Martin Charcot in the nineteenth century after he associated specific patterns of spinal cord white and grey matter damage with patients exhibiting muscle weakness and atrophy [1]. Death normally occurs within 2–3 years of symptom onset, following weakness of the respiratory muscles leading to respiratory failure [2]. The only treatments currently available are riluzole and edaravone, which lead to a modest improvement in lifespan [3, 4]. The majority of ALS patients have no family history of the disease and are classed as sporadic ALS (sALS) patients [5], whereas approximately 5% have a family history of the disease due to inheritance of a mutation in an ALS associated gene and are classed as familial ALS (fALS) patients [6]. A third classification of ALS exists, often referred to as Western Pacific ALS, which occurs with unusually high incidence in a few regions including Guam and the Japanese Kii peninsula, leading to the suggestion that it had an environmental cause [7, 8].

Studies of fALS patients have identified a number of genes which are causally associated with ALS (Table 1), the two most common of which are the chromosome 9 open reading frame 72 (*C9ORF72*) gene [9, 10], and the copper − zinc superoxide dismutase (*SOD1*) gene [11]. Studies on fALS genes have suggested a number of possible mechanisms of motor neuron degeneration including excitotoxicity, oxidative stress, protein aggregation and defects in cell pathways such as autophagy, RNA metabolism, and the DNA damage response (DDR) [12]. The finding that a number of fALS genes play roles in the DDR is particularly striking as DNA damage has been established to be a feature of both sporadic and familial ALS since the 1990s [13, 14], before many of these genes were linked to ALS. DNA damage and deficiencies in the DDR thus may play a widespread role in ALS.

**Table 1:** Key Mendelian genes associated with ALS

Gene	Full Name	Healthy Role	fALS Prevalence	Key Reference
*C9ORF72*	Chromosome 9 open reading frame 72	Autophagy	3–34%	[9, 10]
***SOD1***	**Superoxide dismutase type-1**	**Oxidative stress**	**15–30%**	[11]
***TARDBP***	**Transactive response DNA binding protein 43 kDa**	**RNA metabolism, DDR**	**1–4%**	[19]
***FUS***	**Fused in sarcoma**	**RNA metabolism, DDR**	**3–6%**	[20, 21]
***NEK1***	**Never-in-mitosis A related protein kinase 1**	**Cell cycle, DDR**	**3%**	[22]
*OPTN*	Optineurin	Autophagy	3%	[23]
***SQSTM1 *or** ***p62***	**Sequestosome 1 or p62**	**Ubiquitination, autophagy, DDR**	**2%**	[24]
***VCP***	**Valosin-containing protein**	**Proteasome, vesicle trafficking, autophagy, DDR**	**1–2%**	[25]
*TBK1*	TANK-binding kinase 1	Autophagy	1%	[26]
***SETX***	**Senataxin**	**R loop resolution**	**< 1%**	[27]
*ALS2*	Alsin	Vesicle trafficking	< 1%	[28]
*CHCHD10*	Coiled-coil-helix-coiled-coil-helix domain-containing protein 10	Mitochondrial function	< 1%	[29]
*CHMP2B*	Charged multivesicular body protein 2B	Vesicle trafficking, autophagy, lysosomal pathway	< 1%	[30]
*MATR3*	Matrin 3	Transcription, RNA metabolism	< 1%	[31]
*PFN1*	Profilin 1	Cytoskeleton, axon growth	< 1%	[32]
*UBQLN2*	Ubiquilin 2	Proteasome, autophagy	< 1%	[33]
*VAPB*	Vesicle-associated membrane protein-associated protein B/C	Autophagy	< 1%	[34]

For more comprehensive review of ALS genetics, see [35]. Prevalence of < 1% indicates gene mutations only present in a few families or cohorts, making accurate prevalence measurements difficult. Bold rows indicate genes thought to be involved in the DNA damage response (DDR) and repair

ALS is also considered to have a non-cell autonomous contribution to disease, as glia from ALS patients, including astrocytes, oligodendrocytes and microglia, exhibit a toxic phenotype not observed in healthy cells [15]. Of particular note, ALS astrocytes regardless of disease background, induce cell death in healthy motor neurons both via direct contact and through secreted factors [16], demonstrating non-cell autonomous mechanisms of motor neuron death. While the secreted factors that cause ALS toxicity remain largely unknown, there is growing evidence that proteins involved in DNA damage and DDR impairments, such as p62 and C9ORF72 dipeptide repeat proteins, could be secreted by ALS astrocytes [17, 18].

### DNA damage and response

**Fig. 1:**
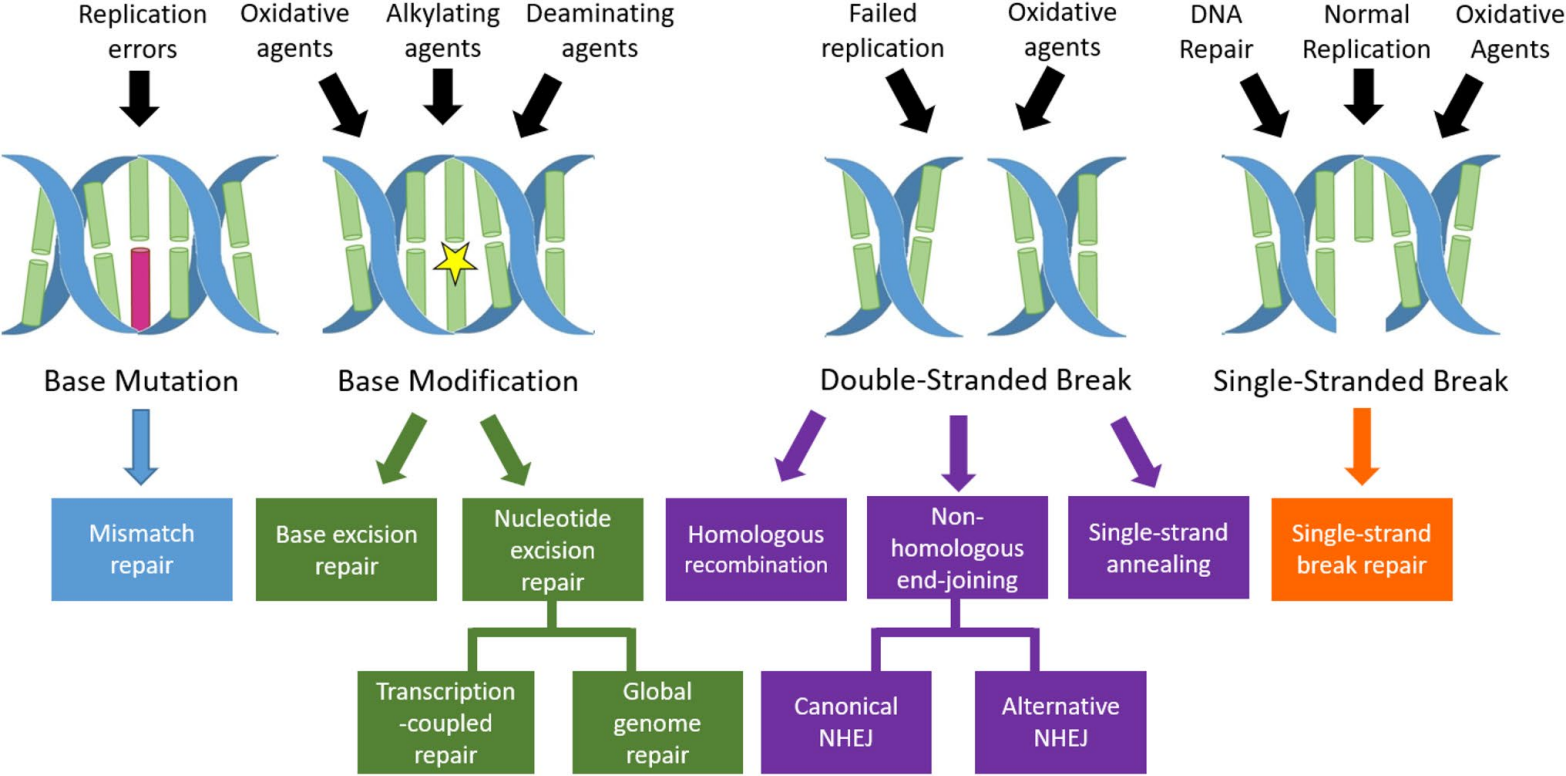
DNA damage and related repair pathways. Various types of DNA damage exist which can be induced by genotoxic agents or can occur during normal cellular events. Several DNA repair pathways exist to repair specific types of DNA damage [36, 37]

DNA damage is a common occurrence in cells, with each cell estimated to experience 10^4^–10^5^ DNA lesions per day. If left unrepaired these lesions can lead to severe consequences, including cell death [38]. DNA damage can occur by chance during transcription or due to harmful genotoxic agents and can affect both nuclear and mitochondrial DNA [37]. Examples of DNA damage (Fig. 1) include oxidation or deamination of bases, base insertions or deletions or substitutions, and DNA double or single-stranded breaks (DSBs or SSBs) [36]. Oxidative DNA damage is measured by assaying for oxidation of DNA nucleosides, usually deoxyguanosine. Oxidised deoxyguanosine (OdG) can exist in two interconverting forms: 8-hydroxy-2-deoxyguanosine (8-OHdG) and 8-oxo-2-deoxyguanosine (8-oxodG) [39]. These names are often used interchangeably in ALS DNA damage studies and will both be referred to as OdG in this article.

### DNA damage response

**Fig. 2:**
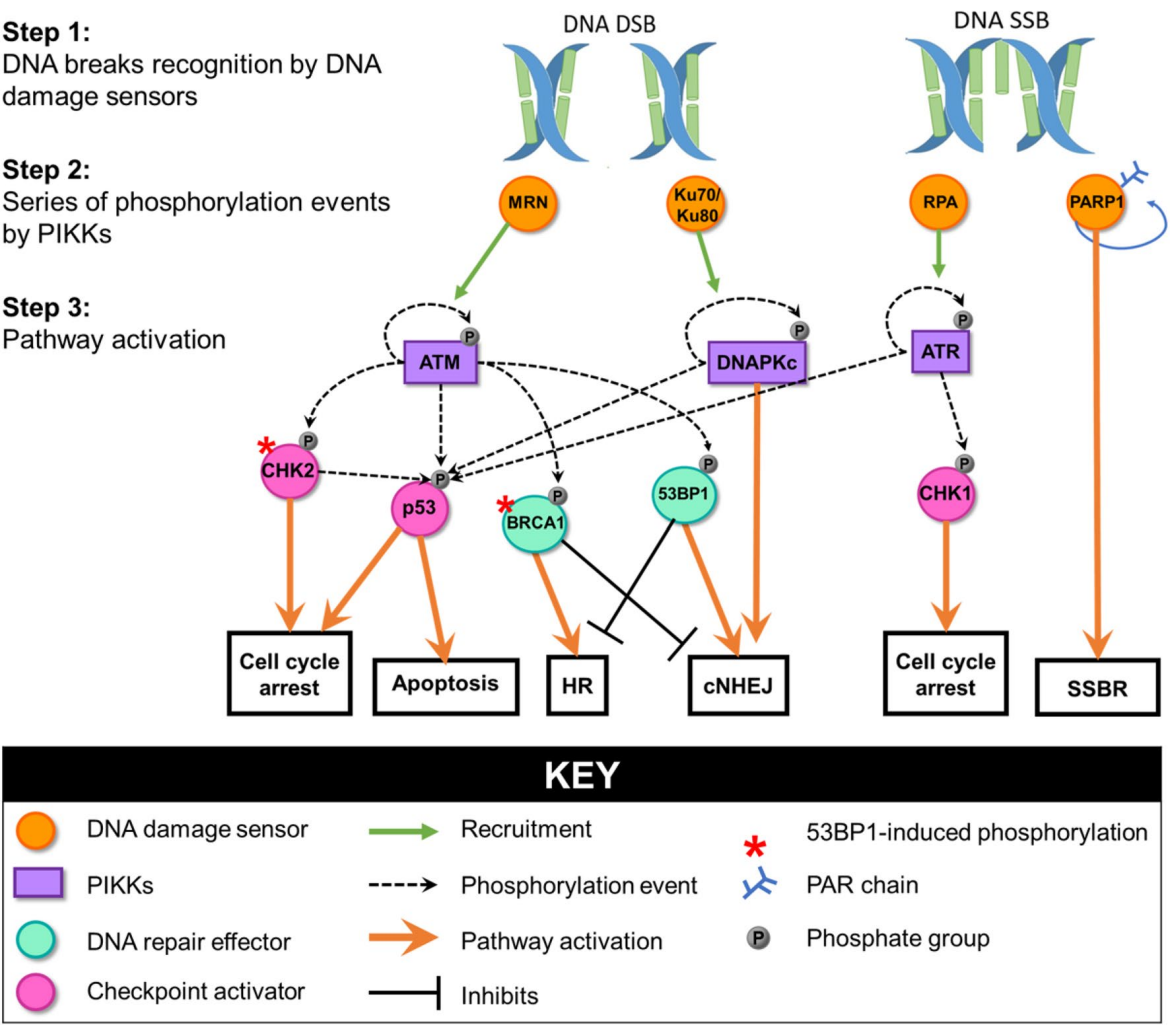
Schematic showing simplified DNA damage response. DNA damage is detected by factors which activate master DNA repair kinases such as ATM and ATR. The repair kinases phosphorylate downstream targets to lead to cell cycle arrest, apoptosis and DNA repair. Notably, several actions including p53 phosphorylation are redundant and performed by multiple kinases [36, 38]

The DNA damage response (DDR) is a signal transduction pathway (Fig. 2) which exists to detect and respond to DNA damage. DNA damage is detected by sensors which bind the DNA ends, including the MRN complex, Ku70/80 heterodimer and RPA [36]. The DNA damage sensors activate master DNA repair phosphatidylinositol 3-kinase-related kinases (PIKKs), including ataxia telangiectasia mutated (ATM) and ATM and Rad3 related (ATR), which induce a phosphorylation cascade that activates effector proteins involved in pathways including cell cycle arrest, chromatin remodelling, DNA repair, and apoptosis [36]. Another key DNA repair kinase is DNA-PK, which is composed of a catalytic subunit (DNA-PKcs) and the Ku70/Ku80 heterodimer. DNA-PK is thought to regulate p53-mediated apoptosis following DNA damage and plays a critical role in non-homologous end-joining (NHEJ) DNA DSB repair by recruiting and phosphorylating NHEJ DNA repair proteins [40]. Two key phosphorylation targets of ATM, ATR and DNA-PK are histone H2AX and p53. Phosphorylated histone H2AX (γH2AX) is thought to act as a docking site for DDR signalling and DNA repair, as several DDR components co-localise with γH2AX foci [38]. p53 is involved in activating DNA repair pathways, but if DNA damage is too extensive or cannot be repaired, then p53 promotes apoptosis by increasing the transcription of pro-apoptotic genes [41].

### DNA damage response and repair in neurons

DNA damage and DNA repair deficiency have long been associated with neurodegeneration as mutations in DDR and DNA repair genes have been found to cause a number of neurodegenerative diseases, including ataxia telangiectasia, ataxia telangiectasia-like disorder, Nijmegen breakage syndrome, ataxia-oculomotor apraxia-1 and 2, ALS4 (caused by mutations in *SETX*, [27]), spinocerebellar ataxia with axonal neuropathy-1, and Cockayne syndrome [42]. DNA damage has also been implicated as playing a role in Alzheimer’s disease, Huntington’s disease and Parkinson’s disease [42]. Notably, the majority of neurodegenerative diseases caused by DDR or DNA repair gene mutations affect cerebellar neurons specifically rather than motor neurons. Motor neurons can also be affected by DNA repair deficiencies as mice with reduced expression of Ercc1, a protein involved in nucleotide excision repair (NER), show age-dependent motor neuron degeneration and astrogliosis, similar to ALS [43].

Despite a clear association between DNA damage and neurodegeneration, the DDR and DNA repair have not been extensively studied in neurons or compared between different neuron populations, and it remains unclear whether specific neuron types like motor neurons are more vulnerable to DNA damage or DNA repair deficiencies. DNA repair kinetics have been compared between astrocytes and neurons, and it was shown that both astrocytes and neurons are dependent on TDP1 for DNA SSB repair [44]. One likely explanation for general neuron vulnerability to DNA damage is their high metabolic activity and reliance on oxidative phosphorylation over glycolysis as their main source of energy, which leads to increased generation of reactive oxygen species and consequently leads to increased oxidative DNA damage [42]. A factor that could compound this effect is the mitotic status of neurons as it has previously been suggested that post-mitotic cells are more likely to accumulate DNA damage than mitotic cells. For example, it has been shown that post-mitotic parenchymal liver cells exhibit an age-related increase in alkali-labile sites that is not observed in mitotically active non-parenchymal liver cells [45].

DNA repair mechanisms differ between mitotic and post-mitotic cells. Some DNA repair mechanisms, specifically homologous recombination (HR) and mismatch repair (MMR), are dependent on the cell cycle and thus may play more of a role in dividing cells compared to post-mitotic cells [46]. Studies have also shown that base excision repair (BER) and nucleotide excision repair (NER), which occur independent of the cell cycle, are affected by mitotic status. Post-mitotic neurons have reduced activity of BER and NER global genome repair (but not transcription-coupled repair), and reduced levels of some BER proteins compared to the mitotic cells they were differentiated from [47, 48]. DNA repair may, therefore, be less efficient in neuronal cells and contribute to neuron vulnerability to DNA damage. Notably, DNA breaks in neurons are not always detrimental. Indeed, neuronal activity has been shown to induce DNA DSB generation by topoisomerase IIβ for the purpose of activating transcription of neuronal activity early response genes, which play roles in synaptic plasticity [49]. These activity-induced DSBs are rapidly repaired through the canonical non-homologous end-joining (c-NHEJ) DNA repair pathway [49], indicating neurons are capable of repairing targeted DNA damage.

DNA damage response and DNA repair have been examined as potential therapeutic targets for neurodegeneration. As such, it is of importance to generate models to test putative molecules. For example, knockout of *tdp1* in zebrafish identified *apex2* and *ercc4* as putative molecules which compensated for *tdp1* loss [50]. Enforcing DNA repair has been shown to improve motor neuron survival following injury. In an in vivo mouse study, expression of human BER proteins, OGG1 or APEX1, attenuated phosphorylated p53 expression in lesioned neurons and reduced motor neuron apoptosis following axotomy, with a greater effect seen with APEX1 expression [51]. APEX1 overexpression has also been shown to improve cell viability following oxidative stress induction in hippocampal neurons or sensory neurons, while APEX1 knockdown reduced cell viability [52]. Surprisingly, suppressing the DDR has also been shown to be neuroprotective as ATM inhibition reduces DNA damage-mediated apoptosis in genotoxin-treated neurons [53]. Similarly, PARP inhibition has been shown to be neuroprotective in models of Huntington’s disease [54] and stroke [55]. It may be that inhibiting DDR signalling prevents p53 phosphorylation and consequently prevents p53-mediated apoptosis. Thus, suppressing the DDR or enforcing DNA repair could be a therapeutic strategy for neurodegenerative disease.

## Specific ALS subtypes and DNA damage

### C9ORF72

**Fig. 3:**
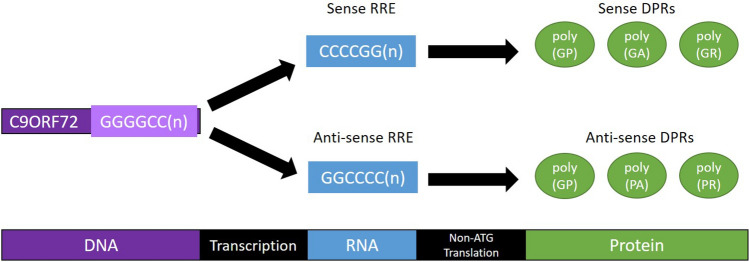
Transcription and translation of the *C9ORF72* repeat expansion. The *C9ORF72* repeat expansion is transcribed in both the sense and anti-sense directions to produce two RNA repeat expansion transcripts. Each transcript can be processed by non-ATG translation to produce a total of five different types of dipeptide repeat protein. The poly(GP) DPR is produced by translation of both the sense and anti-sense transcripts [61]. RRE = RNA repeat expansion, DPR = dipeptide repeat protein

A hexanucleotide (GGGGCC) repeat expansion in the first intron of the chromosome 9 open reading frame 72 (*C9ORF72*) gene is the most common cause of fALS in the West [9, 10]. Several studies have identified increased protein expression and staining for γH2AX in *C9ORF72*-ALS patient post-mortem spinal cord tissue and iPSC-derived motor neurons, suggesting DNA damage is increased in *C9ORF72*-ALS [56–60]. Products of the *C9ORF72* repeat expansion have been suggested as a primary cause of *C9ORF72*-ALS. The repeat expansion is transcribed into sense and antisense repeat-expansion RNAs (RREs), which can undergo repeat-associated non-ATG (RAN) translation to generate five dipeptide repeat proteins (DPRs): poly(GA), poly(GR), poly(GP), poly(PR) and poly(PA) [61] (Fig. 3). Viral expression of *C9ORF72*-ALS RREs or certain DPRs (Table 2) in neuronal cells is sufficient to induce DNA strand breaks and increased γH2AX levels [56, 58, 62], suggesting DNA damage in *C9ORF72*-ALS is caused by RREs or DPRs.

**Table 2:** Summary of DPR properties

DPR Species	Transcript	Toxic to Motor Neurons?	Induces DNA damage?	References
Poly(GA)	Sense	Yes	Yes_1_/No_2_	[56, 59, 62]
Poly(GR)	Sense	Yes	Yes	[56, 58, 62, 63]
Poly(GP)	Sense and anti-sense	No	Not reported	
Poly(PA)	Anti-sense	No	Not reported	
Poly(PR)	Anti-sense	Yes	Yes	[58, 62]

_1_[59, 62] showed transfecting cells with poly(GA) led to increased DNA damage, but _2_[56] did not find the same effect. DPR toxicity or non-toxicity shown in references [64–66]

Increased DNA damage in *C9ORF72*-ALS could be caused by changes in genotoxic agents, such as reactive oxygen species (ROS) [56], or due to increased R-loop formation [59]. ROS are a natural source of DNA damage and have been implicated as the cause of DNA damage in *C9ORF72*-ALS. ROS production has been shown to increase at the same time as DNA damage in *C9ORF72*-ALS iPSC-derived motor neurons and poly(GR) transfected cells, in an age-dependent manner [56]. Additionally, DNA damage could be partly rescued by depleting ROS with an anti-oxidant treatment [56]. A possible cause of increased ROS could be mitochondrial dysfunction, which is known to be induced by poly(GR) expression [63]. Similarly, R-loops, which are naturally occurring RNA:DNA hybrids that can induce DNA strand breaks, are increased in *C9ORF72*-ALS post-mortem spinal cord tissue and poly(GA) DPR transfected cells [59]. Both DNA damage and cell death in poly(GA) transfected cells could be partly rescued by overexpressing senataxin, a gene involved in R-loop resolution [59]. Thus, increased ROS and/or R-loops have been shown to contribute to increased DNA damage in *C9ORF72*-ALS.

Another consequence of the repeat expansion in *C9ORF72*-ALS is the accumulation of protein-linked DNA breaks caused by trapped TOP1 cleavage complexes (TOP1cc), as observed in cells expressing RREs and DPRs [59, 67]. Of relevance, these mechanisms may also feed into each other, as ROS are known to cause both R-loops and TOP1cc [68]. In turn, ROS-dependent accumulation of co-transcriptional R-loops and TOP1cc-dependent SSBs in opposite neighboring DNA strands induces DSBs during transcription [68–71], thus exacerbating a cellular insult that can result in neuronal death.

DDR signalling and DNA repair have been shown to be dysfunctional in *C9ORF72*-ALS and could contribute to increased DNA damage. Repeat expansions in general have been suggested to hinder DNA repair [72]. Furthermore, chromatin compaction and expression of factors that promote this process are increased in *C9ORF72*-ALS spinal cord tissue and poly(GA) transfected cells, which could hinder access to DNA repair factors. Accordingly, inducing chromatin relaxation reduced γH2AX levels and cell death in poly(GA) transfected cells [59].

Interestingly, while poly(GA), poly(GR) and poly(PR) all cause DNA damage [56, 59, 62, 63], they appear to have different effects on the DDR and DNA repair (Table 3). Poly(GA), in fact, has been shown to aggregate and sequester pATM in the cytoplasm, preventing its recruitment to DNA damage sites [62]. This subsequently leads to reduced pATM, 53BP1, and phosphorylated p53 expression in poly(GA) transfected cells, with the effect persisting following DNA damage induction [59, 62]. The significance of this effect remains unclear, however, as *C9ORF72*-ALS motor neurons and cells transfected with poly(GR) or poly(PR) DPRs show an expected increase in pATM, 53BP1 and phosphorylated p53 expression, corresponding with the observed DNA damage [58, 62, 73]. Importantly, p53 appears to play a key role in *C9ORF72*-ALS and DPR-induced toxicity as p53 knockout or knockdown has been shown to extend the lifespan of a mouse model expressing poly(PR), and protect against neurodegeneration in Drosophila models expressing the *C9ORF72* repeat expansion [74]. Strikingly, p53 knockout also reduces DNA damage (γH2AX levels and comet tail measurements) in poly(PR) transduced cells and *C9ORF72*-ALS iPSC-derived motor neurons, indicating p53 action may be occurring upstream of DNA damage rather than downstream [74].

**Table 3:** Effects of DPRs on DDR and DNA repair

DPR	DDR signalling [58, 59, 62, 73, 74]	DNA repair [60]	Binds DDR factors?	Other effects in cells
Poly(GA)	Reduced pATM; reduced 53BP1; reduced p53	Reduced c-NHEJ; reduced SSA	pATM [62]; HR23B [75]	Increased R-loops [59]; increased chromatin compaction (H3K9me3, HDAC4) [59]
Poly(GR)	Increased pATM; increased p53; increased Ku80	Reduced c-NHEJ	NPM1 [58, 60]	Increased ROS [56]; mitochondrial defects [63]
Poly(PR)	Increased pATM; increased p53	Reduced c-NHEJ; reduced SSA; reduced alt-NHEJ	NPM1 [58, 60]	Not reported

The ATM signalling pathway is thought to be primarily involved in homologous recombination (HR) DNA repair [76]. While DPRs have been shown to reduce the efficiency of certain DNA repair pathways (Table 3), they have not been shown to affect HR DNA repair [60]. Instead, DPRs appear to affect canonical and alternative non-homologous end-joining (c-NHEJ and alt-NHEJ) and single-strand annealing (SSA) DNA repair [60]. Most notably, efficiency of c-NHEJ is reduced following transfection with any of the three key DNA damage-inducing DPRs: poly(GA), poly(GR) and poly(PR) [60]. C-NHEJ is mediated through the Ku70/Ku80 DDR pathway [77], which has been shown to be affected in *C9ORF72*-ALS. Ku70, Ku80 and DNA-PKcs expression were shown to be increased in *C9ORF72*-ALS iPSC-derived motor neurons and poly(GR)-expressing Drosophila [73]. Accordingly, knockdown of Ku80 or ATM reduced cell death in this fly model [73]. It was suggested that Ku80 overexpression may lead to NHEJ overactivation [73], which is perhaps unexpected considering the reduced c-NHEJ efficiency observed in the other study [60]. However, it has previously been shown that overexpression of Ku70 and Ku80 in rat fibroblasts leads to reduced DNA-PK activity and increased residual DNA damage twenty-four hours after irradiation [78], suggestive of a NHEJ impairment. Thus increased Ku70/Ku80 signalling could be another mechanism inducing cell death in *C9ORF72*-ALS.

Reduced efficiency of DNA repair could also be mediated through NPM1, a DNA repair factor associated with BER, NHEJ and SSA, as poly(GR) and poly(PR) directly bind and potentially impair NPM1 [58, 60]. Poly(GA) has also been observed to form cytoplasmic inclusions with HR23B, a protein involved in nucleotide excision repair (NER), although NER efficiency was reported to be unaffected [75]. Thus, increased DNA damage in *C9ORF72*-ALS could arise from deficiencies in DNA repair or the DDR, as well as potential increases in genotoxic agents.

## SOD1

Mutations in the copper − zinc superoxide dismutase (*SOD1*) gene were the first identified the cause of fALS [11] and remain the second most common cause of fALS, being responsible for approximately 15% of all fALS cases and 1% of sALS cases [79]. SOD1 is an antioxidant enzyme which protects cells against ROS, so *SOD1* mutations were thought to cause motor neuron death through increased oxidative damage [80]. Limited studies of human *SOD1*-ALS patients have shown increased levels of OdG in the CSF [81, 82], but reduced levels in the motor cortex compared to controls [13]. By contrast, with the exception of one study finding no change in DNA damage [83], studies of SOD1^G93A^ mouse models have consistently shown increased OdG or γH2AX levels, specifically in spinal cord, frontal cortex and striatum, but not the cerebellum which is spared of neurodegeneration [84–87]. Two studies showed DNA damage occurred pre-symptomatically in SOD1^G93A^ mice [85, 86], however, another only observed DNA damage after symptom onset [84]. These results could suggest DNA damage is a cause of motor neuron degeneration and interestingly interventions such as docosahexaenoic acid (DHA) enriched diet which improve *SOD1*-ALS mouse survival have also been shown to reduce DNA damage [88].

Loss of SOD1 function could be a mechanism through which *SOD1* mutations lead to DNA damage. Wild-type SOD1 has been suggested to be protective against DNA damage as two studies have shown cells transfected with SOD1^WT^ exhibit less DNA damage and oxidative stress than untransfected cells before and after H_2_O_2_ treatment [89, 90], although another study failed to replicate this finding [91]. We thus might expect that *SOD1* mutations would lead to the loss of this protective effect. Accordingly, expressing SOD1^A4V^, SOD1^L38V^ or SOD1^G93C^ led to similar DNA damage levels as untransfected cells but higher levels than cells expressing SOD1^WT^, whereas transfection with SOD1^G93A^ or SOD1^H46R^ led to increased DNA damage compared to both untransfected and SOD1^WT^, suggesting an additional gain of function effect for these mutations [89, 91–93].

A likely candidate for the protective effect of SOD1^WT^ against DNA damage would be its dismutase ROS scavenging activity. It would be expected that *SOD1* mutations that lead to increased DNA damage would affect the dismutase activity. This is, however, not the case. Several studies have shown that not all *SOD1* mutations lead to loss of SOD1 ROS scavenging activity [94, 95]. Some mutations only mildly decrease SOD1 activity and others, including the SOD1^G37R^ mutation, lead to increased activity [94, 95]. Indeed, SOD1^A4V^, SOD1^G93A^ and SOD1^L38V^ exhibit dismutase activity levels similar to SOD1^WT^ [95], indicating that if there is loss of a protective function of SOD1^WT^ function in these lines, it is not the dismutase function. On the other hand, the SOD1^H46R^ mutation has been reported to lead to reduced dismutase activity due to severe metal deficiency [95], and while the G93A mutation is thought not to affect dismutase activity [95, 96] it does lead to increased peroxidase activity and a resulting increase in hydroxyl radical production [96]. Thus, changes in SOD1 dismutase or peroxidase activity and subsequent increases in ROS could be a mechanism through which DNA damage is increased in *SOD1*-ALS but is likely not the only mechanism.

Another mechanism through which wild-type SOD1 could exert a protective effect is through an as yet uncharacterised role in the DDR. SOD1^WT^ has been shown to associate with nuclear chromatin and this association is increased in SOD1^G93A^ transfected cells [91]. However, another study showed that expressing SOD1^G93A^ could cause SOD1^WT^ to become sequestered in the cytoplasm. Inducing nuclear import of SOD1 in these cells notably improved cell survival [87], suggesting a protective role for SOD1^WT^ in the nucleus.

In addition to potentially impairing the role of wild-type SOD1 in the DDR, *SOD1* mutations have been shown to affect other DDR processes. Expression of Spy1, a protein that inhibits apoptosis following DNA damage, is reduced in SOD1^G93A^ transfected cells and SOD1^G93A^ mouse spinal cord. Accordingly, overexpressing Spy1 reduced DDR activation and cell death [92]. Reduction in Spy1 thus could sensitise cells to DNA damage and push the balance towards DNA damage rather than DNA repair. Potentially compounding this effect is the finding that expression of wild-type p53-induced phosphatase 1 (Wip1), a protein which dephosphorylates several DDR signalling proteins thus terminating the DDR, is also reduced in *SOD1*-ALS mice and *SOD1*-ALS cell models. Like with Spy1, overexpression of Wip1 improved cell viability [97]. Inability to terminate the DDR could lead to persistent activation and subsequently drive cells towards apoptosis.

The more classical DDR factors may also be involved in *SOD1*-ALS DNA damage. In accordance with the observed damage, DDR activation is increased in cells expressing SOD1^G93A^ as they exhibit increased p53 activity [91], and increased expression of pATM, pATR, pChk1 and p53 [87, 92]. However, it has been shown that several DDR and DNA repair components, including p53, FUS, HDAC1 and APEX1, fail to enter the nucleus or are mislocalised to the cytoplasm in cells expressing SOD1^G93A^ [87]. This was not true for all DNA repair proteins as XRCC1, OGG1 and PARP1 showed normal localisation in SOD1^G93A^-expressing cells [87], and OGG1 has also been shown to have normal nuclear localisation in hSOD1^G93A^ transgenic mice [98]. Cytoplasmic restriction of DNA repair proteins would likely render them functionally inactive and potentially prevent DNA repair processes [87], however, it should be noted that several other studies have failed to observe mislocalisation of DNA repair proteins. Increased nuclear p53 immunoreactivity has been observed in spinal motor neurons in SOD1^G86R^ mice [99]. Similarly, APEX1 is upregulated and enriched in the nuclei of ALS patient motor neurons [100], however, the majority of these patients were classed as sporadic so it is unclear if this also applies to *SOD1*-ALS patients. One study in SOD1^hG93A^ mice found that expression of APEX1 was reduced in spinal motor neurons pre-symptomatically, indicating that a deficiency in DNA repair precedes motor neuron degeneration [101]. SOD1^G93A^ expression in cells deficient in the DDR protein aprataxin has been shown to sensitise cells to oxidative stress, exacerbate DNA repair deficiencies and increase levels of heterochromatin [102]. On the other hand, DNA repair has been suggested to be unaffected in *SOD1*-ALS iPSC-derived motor neurons as these motor neurons exhibit similar γH2AX kinetics to control motor neurons over time following DNA damage induction [103]. Notably, this shows overall DNA repair kinetics, and it remains unknown whether there are any deficits in specific DNA repair pathways, such as NHEJ, which are potentially compensated for. Thus, changes in expression or localisation of DDR factors may affect DNA repair and play a role in motor neuron degeneration in *SOD1*-ALS.

## FUS

Fused in sarcoma (*FUS*) is an RNA binding protein involved in the DDR, DNA repair and RNA processing, transcription and translation [104], which was also found to be a fALS gene [21]. DNA damage, measured by γH2AX or DNA strand breaks, is increased in *FUS*-ALS post-mortem motor cortex and spinal cord [105, 106], *FUS*-ALS iPSC-derived neural progenitor cells and motor neurons [106, 107], and FUS^R521C^ mice [108]. Given the role of FUS in DDR signalling and DNA repair, it seems likely that DNA damage in *FUS*-ALS is caused by *FUS* mutations affecting these processes.

FUS has been shown to be an early DDR signalling player, being recruited to DNA DSBs by PARP1 [105, 109–111]. The presence of FUS at chromatin is sufficient to induce γH2AX foci formation and may recruit other factors as FUS knockdown leads to reduced pATM retention at DNA DSBs [105]. *FUS*-ALS iPSCs exhibit a greater increase in γH2AX following irradiation than control iPSCs, indicating an increased sensitivity to DNA damage [107]. *FUS*-ALS mutations do not affect FUS accumulation at DNA DSBs, instead they affect recruitment of factors, as the presence of pATM and HDAC1 at DNA DSBs was reduced in cells expressing mutant FUS [105]. Another characteristic of mutant FUS is its propensity for cytosolic mislocalisation [107]. Motor neurons expressing mutant FUS were shown to form cytoplasmic FUS-bearing stress granules. The severity of the mutation was also shown to be associated with greater amounts of mislocalised FUS and with earlier disease onsets [107]. Whether FUS mislocalisation to the cytoplasm influences early DDR signalling is unknown. Interestingly, HDAC inhibitors, which reduce mutant FUS mislocalisation to the cytoplasm, have been shown to improve recruitment of FUS^P525L^ to DNA damage sites, potentially preserving DDR signalling and DNA repair and thus suggesting FUS mislocalisation impacts DNA repair [112].

The role of FUS in later DDR signalling events is contested. Some studies have suggested FUS is not necessary for signalling downstream of ATM, as FUS knockdown does not affect levels of phosphorylated 53BP1, phosphorylated Chk2 [109], or γH2AX [110]. However, another study showed ATM signalling was defective in FUS-depleted neurons as following genotoxin treatment there was increased DNA DSBs, but reduced levels of γH2AX, 53BP1 foci, and phosphorylated Chk2 [105]. The effect of *FUS*-ALS mutations on the expression of these DDR components has not yet been investigated, but this may be another mechanism through which *FUS*-ALS mutations could lead to increased DNA damage.

As well as the DDR, FUS is also involved in multiple DNA repair processes. FUS knockdown reduces the efficiency of HR and NHEJ [105, 109]. Thus, we might expect *FUS*-ALS mutations to affect these DNA repair processes. Cells expressing ALS-associated mutant FUS exhibited reduced HR efficiency, with the effect dependent on the mutation. Some FUS mutants but not others additionally showed reduced NHEJ efficiency [105]. Reduced DNA repair efficiency is likely due to changes in the expression of DNA repair factors. Motor neurons carrying FUS^P525L^ mutations show reduced protein levels of BRCA1, DNA ligase IV, RAD23B and MSH2; which is also observed in FUS-depleted cells [113]. DNA damage in *FUS*-ALS is likely to arise from loss of function effects on DNA repair.

## TDP-43

The transactive response DNA binding protein 43 kDa (TDP-43) was first associated with ALS as a pathological marker. In sALS, *C9ORF72*-ALS and many other fALS variants (but not *SOD1*-ALS), TDP-43 is mislocalised from the nucleus to the cytoplasm, and can be found in ubiquitin-positive inclusions [114, 115]. It was only later that mutations in the TDP-43 gene, *TARDBP*, were found to cause a subset of ALS cases [19]. Therefore, when considering the potential role of TDP-43 in DNA damage, it is likely to apply to not only *TARDBP*-ALS cases but potentially sALS and fALS as well. An initial study of *TARDBP*-ALS iPSC-derived motor neurons (carrying S393L and G294V mutations) showed reduced survival but no change in γH2AX, suggesting DNA damage was not a feature of *TARDBP*-ALS [116]. However, more recent studies have shown increased γH2AX in the spinal cord of a *TARDBP*-ALS patient carrying the Q331K mutation [117] and in fibroblasts from a *TARDBP*-ALS patient carrying the M337V mutation [118]. Additionally, γH2AX has also been shown to be increased in cortical neurons of a mouse model of TDP-43 mislocalisation [118]. It is possible that increased DNA damage in *TARDBP*-ALS is mutation dependent, however, there is considerable evidence suggesting the involvement of TDP-43 in DNA damage response and repair that could be affected by *TARDBP* mutations.

TDP-43 was shown to play a role in DDR signalling by associating with several DDR proteins [119, 120]. Following DNA damage induction by transcriptional arrest or DNA DSB generation, TDP-43 colocalises with γH2AX and FUS in the nucleus [111, 118], and can directly bind DNA DSBs [119]. Additionally, TDP-43 interacts with components of the NHEJ protein complex, with the association increasing following DNA damage induction [119]. TDP-43 further aids NHEJ-mediated DSB repair by promoting the recruitment of XRCC4-Lig4 complex to the chromatin [119]. Interestingly, TDP-43 containing ALS-linked mutations, including the A315T or Q331K mutations, is still recruited to DNA damage sites but shows reduced interaction compared to wild-type TDP-43 [118]. Accordingly, overexpressing wild-type TDP-43 led to reduced DNA damage following etoposide treatment in NSC-34 cells, with the protective effect lost when TDP-43 carrying the Q331K or A315T mutations was expressed instead [118]. TDP-43 mutations thus may lead to impairment of the function of TDP-43 in DDR signalling or DNA repair.

In fact,TDP-43 knockdown has been shown to lead to increased DNA strand breaks [111, 119] but no increase in γH2AX [118]. One study suggested this only occurred in response to the transcriptional arrest, and DNA damage could be rescued by overexpressing a protein that resolves R-loops, suggesting TDP-43 may be involved in the prevention or repair of R-loop associated DNA damage [111]. It thus might be expected that R-loops would be increased in *TARDBP*-ALS, however, this has not yet been investigated. Long-term TDP-43 depletion has been shown to lead to a long-term increase in pATM, indicating sustained DDR activation, and eventually cell death [119]. In addition,TDP-43 depletion leads to reduced association of NHEJ DNA repair proteins XRCC4, Lig4 and XLF with γH2AX and 53BP1 [119]. This results in an overall reduction in NHEJ activation following induction of DNA DSBs in TDP-43 depleted cells [119]. Similar results were seen in cells expressing TDP-43 carrying the Q331K or A315T mutations and in fibroblasts from a *TARDBP*-ALS patient carrying the M337V mutation, with a specific impairment in the c-NHEJ pathway observed rather than the alt-NHEJ pathway [118]. This way, TDP-43 mutations or mislocalisation could lead to increased DNA damage through reduced DNA repair or impaired DDR signalling.

## NEK1

Never-in-mitosis A related protein kinase 1 (*NEK1*), another gene associated with the DDR [121], is also a fALS gene [22]. As *NEK1* was only recently associated with ALS, there is only one associated DNA damage study. *NEK1*-ALS iPSC-derived motor neurons showed increased γH2AX compared to controls, indicating DNA damage is a feature of *NEK1*-ALS [57]. Like with FUS, it is likely that DNA damage in *NEK1*-ALS is due to the haploinsufficiency of NEK1 affecting its involvement in DDR signalling and DNA repair. NEK1 protein expression is reduced by 50% in *NEK1*-ALS patient cells [57], and NEK1 knockdown has also been shown to lead to increased morphological signs of DNA damage [122] and reduced cell survival following genotoxic treatment [121, 123]. Thus, NEK1 depletion leads to increased DNA damage and increased sensitivity of cells to DNA damage.

NEK1 may play a role in DDR signalling downstream of ATM/ATR. At baseline and following DNA damage induction, *NEK1*-ALS iPSC-derived motor neurons do not exhibit changes in pATM levels but do exhibit elevated pBRCA1 and slightly reduced p53 [57]. This could mean *NEK1*-ALS motor neurons are more ‘primed’ to respond to DNA damage. NEK1 is also involved in cell cycle arrest. Following DNA damage induction, both *NEK1*-ALS motor neurons and NEK1 knockdown cells exhibit reduced phosphorylation of Chk1 and Chk2 which would potentially prevent cell cycle arrest [57, 123, 124]. Cell cycle re-entry has been suggested to be necessary for DNA repair in neurons [125], thus cell cycle impairment may lead to DNA damage accumulation in *NEK1*-ALS. Interestingly, unlike *NEK1*-ALS motor neurons, actively dividing *NEK1*-ALS iPSCs do not exhibit increased DNA damage compared to controls [57]. Thus, the role of NEK1 may be more important in post-mitotic cells, meaning motor neurons would be more vulnerable to mutations than dividing cells.

NEK1 may also play a key role in DNA repair. Following DNA damage induction, *NEK1*-ALS motor neurons and NEK1 knockdown cells exhibit accumulation of γH2AX over time, indicating a lack of DNA repair [57, 123, 124]. NEK1 has been shown to phosphorylate and activate Rad54, a protein involved in HR DNA DSB repair [126]. Thus, it seems likely that increased DNA damage in *NEK1*-ALS could arise due to deficiencies in HR DNA repair, but this has not yet been investigated.

### Sporadic ALS

DNA damage is not unique to fALS, in fact the first studies reporting DNA damage in ALS were performed in sALS patient post-mortem tissue. They found increased OdG levels in sALS spinal cord and motor cortex, but not in the parietal cortex or cerebellum, suggesting DNA damage was a feature in sALS and was specific to regions where motor neurons degenerate [13, 14, 103]. Without a genetic link to investigate, it has been more difficult to elucidate the mechanisms of DNA damage in sALS motor neurons. Motor neurons are post-mitotic and not replaced throughout life, so exposure to genotoxic agents and/or chance accumulation of DNA damage over time could lead to motor neuron degeneration in sALS. ALS incidence and DNA damage levels are known to increase with age [81, 127], while DNA repair efficiency decreases [128]. Additionally, several suggested risk factors for developing ALS, including smoking and exposure to chemicals, pesticides and metals, could be sources of genotoxic agents [129].

Deficiencies in DNA repair in ALS could be due to changes in the expression of proteins involved in DDR. Consistent with this hypothesis, in sALS motor neurons PARP expression is reduced [130], while phosphorylated c-Abl and BRCA1 expression is increased [103]. In sALS post-mortem motor cortex, APEX1 expression was reported to be reduced in one study [131], but another study showed nuclear enrichment and increased APEX1 activity [100]. Furthermore, methylation of DNA repair genes including OGG1, which is involved in oxidative DNA damage repair, is reduced in sALS motor cortex [103]. Notably mitotic cells, such as bone marrow mesenchymal stem cells and blood cells do not exhibit increase in DNA damage in sALS [132, 133], possibly because of their ability to repair DNA damage during the cell cycle or because of their turnover. This was found not to be true for sALS dermic fibroblasts, which exhibit increased DNA damage and reduced DNA repair, but also have a reduced proliferation rate compared to control fibroblasts, which could account for the increased DNA damage [134]. Mitotic status therefore may affect DNA damage in ALS.

Increased OdG levels have also been observed in the CSF, urine, blood plasma and blood serum of sALS patients [81, 82, 135–137], indicating DNA damage is not just a feature of end-stage of disease. One study suggested levels of DNA damage relate to disease progression, as urine OdG levels correlate negatively with disease progression [81], however, this could not be replicated [136]. Similarly, CSF OdG levels positively correlate with disease duration but not disease severity score [135]. It may be that DNA damage accumulates over time in ALS but does not directly relate to disease progression.

### Western Pacific ALS

Western Pacific ALS, which occurs primarily in Guam and the Japanese Kii peninsula, is clinically very similar to classical sporadic and familial ALS but is suspected of having an environmental cause (reviewed in [8, 138]). Like with sALS and fALS, there is some evidence that DNA damage may be involved in motor neuron degeneration in Western Pacific ALS. A decline in the incidence of Western Pacific ALS was associated with reduced use of traditional foods or medicines containing material from local cycad plants [139]. Cycad seeds contain neurotoxins, including methylazoxymethanol (MAM), β-N-methylamino-L-alanine (BMAA) and β-sitosterol β-d-glucoside [140–142]. It remains debated which, if any, of these toxins causes Western Pacific ALS, but each induces motor impairment and/or motor neuron abnormalities when administered to animals [138, 143, 144]. Interestingly, MAM treatment increases expression of alkylation DNA damage markers in rat cortical neurons and mice, such as *N*^7^-methyldeoxyguanosine and *O*^6^-methyldeoxyguanosine [145, 146], and BMAA treatment increases γH2AX expression in primary human neurons [147] and genomic instability in human blood cells [148]. Taken together, this could suggest cycad neurotoxins induce motor neuron degeneration and ALS-like symptoms through DNA damage. However, there are arguments against the cycad hypothesis of Western Pacific ALS (reviewed in [149]), and it remains unproven whether cycad toxins cause Western Pacific ALS and consequently whether DNA damage may be the mechanism involved.

## Link between defects in protein degradation and DNA damage in ALS

Protein misfolding and aggregation is a hallmark of ALS [151]. The presence of insoluble inclusions containing misfolded proteins increases during the course of the disease, thus indicating defects in protein degradation [152]. Several genes associated with fALS encode for proteins that misfold and aggregate into ubiquitinated inclusions within motor neurons [151]. This is the case of SOD1 [153], TDP-43 [154] and FUS [107]. In addition, *C9ORF72* expansions cause unusual RAN translation that lead to the formation of DPRs, which also accumulate into toxic aggregates [61, 151]. However, this is not exclusive to the familiar forms of ALS since aggregates of ubiquitinated proteins are also present in sALS [155].

The accumulation of misfolded proteins in ALS motor neurons is suggestive of deficient protein degradation mechanisms [156–158]. Consistently, upregulation of protein degradation mechanisms has been successful in clearing toxic aggregates of TDP-43 and FUS [159, 160], as well as SOD1-containing inclusions [161]. This is not surprising given that ALS can arise from mutations in genes encoding for proteins involved in degradation mechanisms, including autophagy and/or in the ubiquitin proteasome (UPS) system (Table 1).

Sequestosome 1 (SQSTM1) or p62 is a scaffold protein involved in numerous pathways. P62 was first described by its role as an autophagic receptor [162]. Via its ubiquitin-associated (UBA) domain, p62 recognizes and binds to ubiquitinated substrates and delivers them to autophagososomes through binding to LC3 mediated by its LC3-interacting region (LIR) [163]. P62 is also involved in the degradation of misfolded proteins through the UPS. Through interaction of its PB1 domain to the 26 s proteasome, p62 facilitates the proteasomal degradation of polyubiquitinated cargos [163]. The presence of inclusions containing p62 has been observed in both fALS and sALS motor neurons, indicating a possible role of p62 in the pathogenesis of the disease [59, 164–166]. In fact, around 2% of fALS patients carry mutations in the *SQSTM1/p62* gene [167].

P62 is itself degraded by autophagy, consequently, impairment of autophagy causes p62 accumulation [162]. Accumulation of p62 has been shown to negatively impact DNA repair in *C9ORF72*-ALS models [59, 67]. The LIM-binding (LB) domain of p62 was shown to interact with the MIU1 domain of RNF168, an E3 ubiquitin ligase responsible for ubiquitinating histone H2A at lysine 15 during DDR [168]. This histone modification signals for the recruitment and stabilization of 53BP1 at the chromatin, thus promoting NHEJ repair [169]. Binding of p62 inhibits RNF168 activity, resulting in defective 53BP1 foci formation [168]. Accordingly*, C9ORF72*-ALS cell models presented defects in 53BP1 signaling, together with a lack of H2A ubiquitination and ATM phosphorylation. These findings suggested p62 involvement and, indeed, p62 ablation restored 53BP1 recruitment [59]. This indicates DNA repair defects and consequent accumulation of DNA damage in ALS could be the consequence of impaired autophagy mechanisms.

In addition, p62 was also found to form cytoplasmic ubiquitin-positive inclusions with TDP-43 in brains from frontotemporal dementia (FTD) patients, indicating p62 is involved in the degradation of misfolded TDP-43 [166]. The LIR domain of p62 is crucial to clear TDP-43 inclusions since the removal of LIR domain resulted in a build-up of TDP-43 aggregates [170]. In support of these findings, L341V and D337E mutations in the LIR domain of p62 have been identified in ALS patients [171]. It is possible that these mutations contribute to ALS pathogenesis by promoting TDP-43 aggregation into cytoplasmic inclusions. TDP-43 mislocalisation and aggregation have a negative impact on DDR [172]. Therefore, p62 mutations could also indirectly interfere with the role of TDP-43 as a DDR player, thus feeding into the DNA repair defects in ALS and further promoting the accumulation of unrepaired damage.

Additional to its role in protein degradation, p62 is also involved in regulating oxidative stress response [162]. Under oxidative stress, p62 binds to Keap1 through its Keap1-interaction region (KIR) [173]. This interaction frees Nrf2 from the inhibitory interaction with Keap1, thus promoting Nrf2 translocation to the nucleus where it acts as a transcription factor for the expression of antioxidant genes [174]. P62^P348L^ and p62^G351A^ mutants were found in ALS patients and affect KIR domain of p62. These mutations interfere with p62 ability to bind to Keap1 and thus exhibit reduced Nrf2 activity [175]. Moreover, two p62 mutations found in FTD patients, A381V and K238del, were associated with defects in mitochondrial membrane potential and limited mitochondrial substrates [176]. It is likely these mutations contribute to ROS accumulation and consequent increase in oxidative stress due to the absence of Nrf2 protective effect. In fact patient cells carrying A381V and K238del mutations exhibit increased ROS production and concomitant with aggravated oxidative stress [176].

Another protein involved in both UPS and autophagy mechanisms is the valosin-containing protein (VCP) or p97, a member of the AAA + family of proteins [177]. Additionally, VCP also promotes NHEJ repair signaling by facilitating the binding of 53BP1 to the histone mark H4K20 after removing the Polycomb protein L3MBTL1 from the chromatin [178]. Mutations affecting VCP have also been identified in ALS patients [179, 180]. The R155H mutant was found to cause defects in autophagosome maturation. Furthermore, this mutant was found to induce TDP-43 translocation to the cytoplasm, leading to the formation of ubiquitinated TDP-43-positive inclusions [179, 181]. Another VCP-ALS mutant, the R159H, was also found to promote the formation of aggregates containing p62 and TDP-43 [179]. As suggested for p62-mediated ALS pathogenesis, it is likely that *VCP*-ALS mutations might interfere with TDP-43 function as a DDR factor, causing defective DNA repair and accumulation of DNA damage. Additional ALS-causing mutations were found to interfere with the ATPase activity of VCP [67, 182], which is crucial for its activity in DDR [183]. The functional relevance of these mutations in the context of the DDR role of VCP is still unknown, but it is possible these VCP mutations cause ALS, in part, by triggering DDR defects and leading to DNA damage accumulation.

Thus, defects in protein degradation mechanisms are linked to the DNA repair defects observed in ALS. This suggests increased DNA damage might be a consequence of the increased protein misfolding and aggregation characteristic of ALS pathology. However, the fact that several ALS-causing mutations affect proteins directly or indirectly involved in DNA repair, thus leading to accumulation of DNA damage, indicates that DNA damage could be a direct cause for motor neuron degeneration. Likely, the accumulation of DNA damage is a combination of cause and effect, both involved in the pathogenesis of ALS.

## Astrocytes and ALS

### Astrocytes and brain function

Astrocytes are the most abundant cell type in the brain and are proposed to have a number of roles in promoting neuron activity. Astrocytes can regulate blood flow to the brain in response to changes in neuron firing, modulate synaptic transmission by secreting glutamate, and take up glutamate to prevent toxic accumulation [184]. Similarly to microglia, astrocytes are thought to exist in two states: a normal ‘resting’ state, and an activated ‘reactive’ state. Reactive astrocytes are thought to be activated following injury to the central nervous system, and respond to injury by becoming phagocytic to clear debris and dead cells, releasing factors to promote neuron survival and helping with scar formation to isolate the site of injury or infection, or repair the blood–brain barrier [185]. Importantly, reactive astrogliosis is a key pathological feature of ALS [186, 187], indicating an important role for astrocytes in ALS pathogenesis.

### Astrocyte toxicity and ALS

Astrocyte toxicity in ALS is the most studied of the toxic interactions between glia and neurons in this disease and it appears consistent across sALS and fALS. Astrocytes were first suggested to be involved in *SOD1*-ALS, as selective astrocyte knockdown of mutant SOD1 in a SOD1 mouse model delayed disease progression and extended survival [188]. It has since been shown that co-culturing motor neurons with astrocytes from sALS, *C9ORF72*-ALS and *SOD1*-ALS patients, as well as from *SOD1*-ALS and *FUS*-ALS mouse models induces neurodegeneration [16, 18, 189–195]. ALS astrocytes have been suggested to be specifically toxic to motor neurons as they do not induce neurodegeneration in GABAergic or dorsal root ganglion neurons [16, 189–191].

The exact mechanisms by which ALS astrocytes induce motor neuron death remain unclear, however, it is clear that astrocyte secreted factors play a major role, as the application of ALS astrocyte conditioned media alone is sufficient to induce neuron death [16, 18, 189–191, 193]. Secretion of extracellular vesicles has been suggested as a vehicle for the delivery of toxic compounds as the application of *C9ORF72*-ALS or *SOD1-*ALS astrocyte exosomes is sufficient to induce motor neuron death [196, 197]. *C9ORF72*-ALS exosome toxicity was partly attributed to microRNAs in the exosomes [196], which is likely specific to the *C9ORF72*-ALS subtype as expression profiling of exosomal microRNAs from SOD1^G93A^ mouse astrocytes showed no significant changes compared to wild-type [195]. SOD1 and TDP-43 protein have also been detected in exosome fractions of cells expressing human SOD1 or TDP-43, respectively [198, 199], suggesting exosomes may also allow the transmission of pathological ALS proteins.

### Astrocytes and DNA damage

**Fig. 4:**
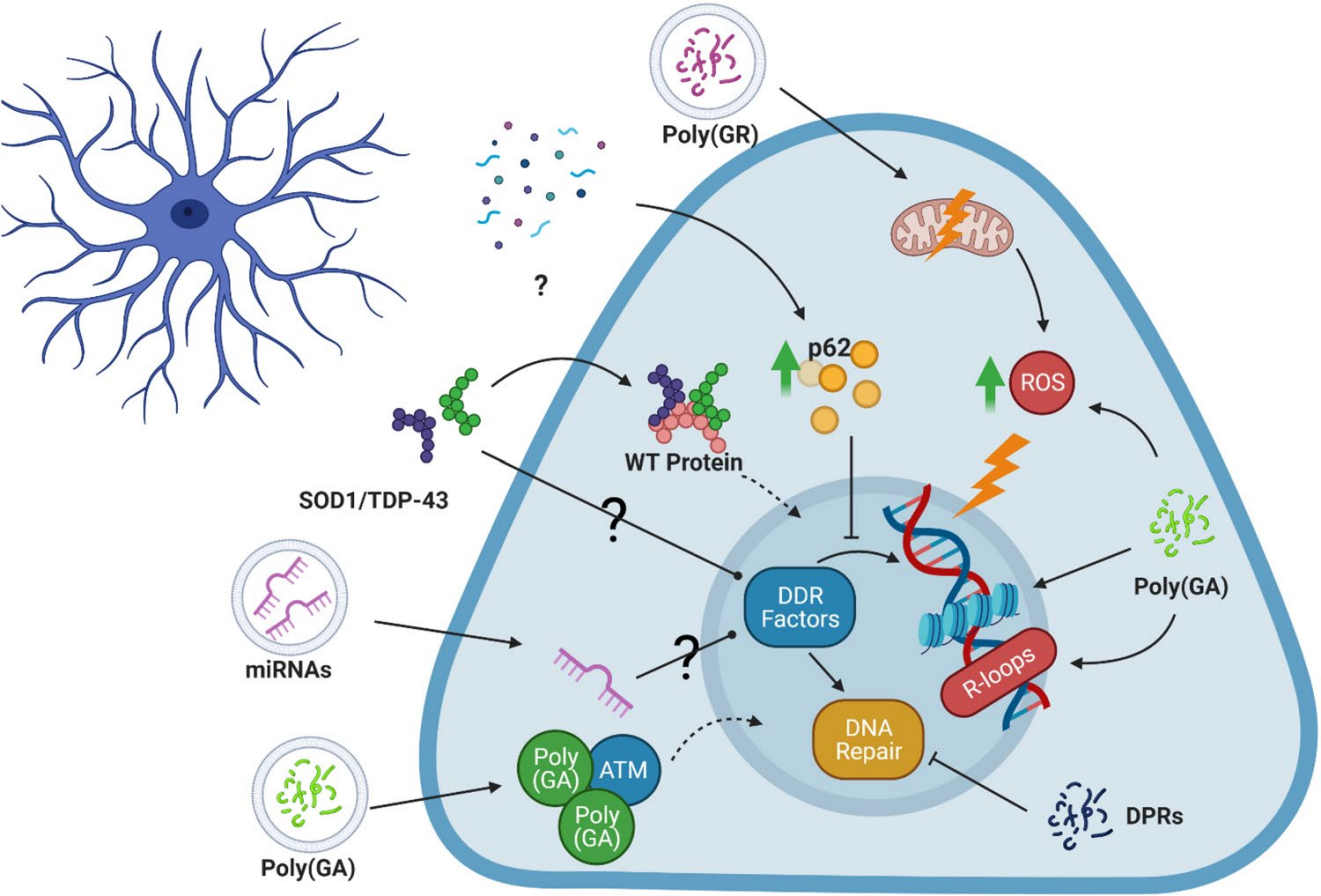
Possible mechanisms for ALS astrocyte-induced DNA damage (Created with Biorender.com). ALS astrocytes secrete various factors that could induce DNA damage in motor neurons. DPRs secreted by *C9ORF72*-ALS astrocytes could sequester DDR factors, induce increases in reactive oxygen species and R-loops and induce chromatin compaction. ALS astrocytes are known to induce p62 accumulation in neurons, which could consequently interfere with DDR recruitment to DNA damage. Transmission of pathological proteins like TDP-43 and SOD1 could sequester wild type protein and potentially affect the DDR. Similarly, microRNAs transmitted by ALS astrocytes could affect DDR factors

While no studies have directly looked at whether astrocytes contribute to DNA damage in ALS, there are some indications that their toxicity to motor neurons could be related to DNA damage (Fig. 4). ALS astrocyte conditioned media has been shown to induce p62 accumulation in motor neurons, concomitant with autophagy impairment [18]. While p62 is primarily known for its involvement in autophagy, it is also a negative regulator of the DDR through its inhibition of the E3 ligase, RNF168, which ubiquitinates histone H2A following DNA damage [168]. As covered in a previous section, histone ubiquitination is needed for DDR factor recruitment, thus astrocyte-induced p62 accumulation could affect DDR factor recruitment to sites of DNA damage and consequently reduce the efficiency of DNA repair [59].

Autophagy deficiency caused by ALS astrocyte conditioned media could also lead to increased misfolding of pathological proteins and contribute to the depletion of important DDR factors, such as TDP-43. TDP-43 expression does not appear affected by ALS astrocyte conditioned media treatment [18], but this does not discount the possibility of TDP-43 mislocalisation or aggregation. Indeed wild-type reactive astrocytes, which behave similarly to ALS astrocytes, induce TDP-43 and SOD1 inclusions in motor neurons [200]. SOD1 expression has also been shown to be increased in cells treated with ALS astrocyte conditioned media, which could not be rescued by autophagy activation, indicating deficient autophagy is not responsible for astrocyte-induced SOD1 expression changes in motor neurons [18].

In addition to inducing protein misfolding through autophagy impairment, ALS astrocytes may also directly transmit pathological proteins to motor neurons. Natively folded and misfolded SOD1 have been shown to be transmitted intercellularly by SOD1-expressing neuronal and non-neuronal cells through exosomes [198]. This is also true of *SOD1*-ALS astrocytes as mutant SOD1 has been detected in both exosome-enriched and exosome-depleted fractions of mouse SOD1^G93A^ primary astrocyte conditioned media [197]. Notably, despite SOD1^G93A^ astrocytes secreting less total protein than wild-type astrocytes, SOD1^G93A^ astrocytes secrete higher levels of SOD1 and intriguingly, VCP [197]. Application of SOD1^G93A^ primary astrocyte exosomes has been shown to lead to transmission of SOD1 to motor neurons and motor neuron death [197]. As discussed previously, there is evidence that expression of mutant SOD1 in motor neurons leads to DNA damage and DDR impairment, which would be exacerbated by astrocyte transmission of SOD1.

SOD1 is not the only pathological protein that may be transmitted by ALS astrocytes. TDP-43 has been detected in the exosome fractions of neuron-like cells expressing wild type or mutant TDP-43, and in exosome fractions from healthy mouse primary neurons, but not astrocyte or microglial exosome fractions [199]. This indicates ALS astrocytes may not transmit TDP-43, however, as the study used healthy mice it is unclear whether the same would occur under disease conditions. DPRs may also be transmitted by *C9ORF72*-ALS astrocytes, which have been shown to express poly(GP) DPRs [201], and likely express other DPRs although this has not yet been shown. It has previously been shown that DPR-expressing motor neurons can transmit DPRs to non-expressing cells, including astrocytes, through both exosome dependent and independent pathways [17]. Although DPR transmission from astrocytes to motor neurons has not been demonstrated yet, this process could exacerbate the existing DPR burden in motor neurons and lead to further DPR-induced DNA damage and DDR dysfunction.

MicroRNAs transmitted by astrocytes could also influence DNA damage and DDR signalling in motor neurons as some species of microRNAs are involved in the promotion or inhibition of DDR signalling and/or DNA repair [202]. Some of the microRNAs identified as dysregulated in *C9ORF72*-ALS astrocyte exosomes [196] have been predicted to target proteins involved in DDR and DNA repair pathways, including miR-140 (NHEJ), miR-200 (cell cycle), miR-494 (transcription-coupled NER), and miR-758 (HR) [202]. miR-494, which is downregulated in *C9ORF72*-ALS astrocytes and extracellular vesicles [196], has also been shown to repress the expression of nucleolin [203]. Nucleolin is an RNA binding protein that plays a number of roles within the cell, including a role in DDR signalling and DNA DSB repair [204]. It would be predicted that miR-494 downregulation in *C9ORF72*-ALS astrocyte exosomes would lead to increased nucleolin expression in motor neurons. Increased nucleolin has previously been shown to confer increased DNA repair activity [205]. On the other hand, poly(GR) and poly(PR) DPRs co-localise with nucleolin and have been suggested to interfere with some nucleolin functions [65, 206, 207]. An increase in nucleolin could potentially enhance recruitment of DPRs to the nucleus and facilitate the DNA damage they induce. Notably, RNA interference of nucleolin increased the viability of poly(GR)-expressing Drosophila [207]. Thus microRNAs secreted by ALS astrocytes may affect DNA damage signalling and repair in motor neurons. It is, however, worth remembering that microRNAs play a number of varied roles, and while we have linked some ALS astrocyte microRNAs to the DDR further studies would have to be conducted to prove these links.

### DNA repair in astrocytes

Like all cells, astrocytes can be subjected to DNA damage and have mechanisms in place to repair the damage. Interestingly, healthy astrocytes have been shown to exhibit reduced DDR signalling compared to other cell types. Expression of the DDR factors ATM, ATR, MRE11, MDC1, CHK2 and p53 are reduced in terminally differentiated astrocytes compared to neural stem cells [208]. Consequently, astrocytes show limited pATM foci formation and no detectable 53BP1 foci following DNA damage induction by irradiation [208]. Despite this reduced DDR signalling, astrocytes still show normal γH2AX foci formation, which appears to be in part due to phosphorylation by DNA-PK [208]. ALS astrocytes are also capable of γH2AX foci formation, with comparable levels of γH2AX observed when comparing *C9ORF72*-ALS patient iPSC-derived astrocytes [56] and primary astrocytes from embryonic *SOD1*-ALS mice [83] to healthy control astrocytes.

Despite astrocyte deficiencies in normal DDR signalling, they are highly radioresistant and capable of repairing DNA damage. Following DNA damage induction, astrocytes exhibit increased expression of the NHEJ factors, Ku70 and XRCC4, and increased expression of the HR factors, RAD51 and RPA [209]. Despite this, like neurons, astrocytes show an age-related increase in DNA damage, indicating a reduced capacity to repair DNA with age [210]. BER activity has been shown to be reduced in aged astrocytes compared to young astrocytes, however, this is not unique to astrocytes and has also been observed in neurons [210]. Similarly, both neurons and astrocytes have been shown to have reduced NER capabilities compared to fibroblasts indicating general CNS cells may have different DNA repair capabilities and mechanisms compared to non-CNS cells [211]. There is some evidence that among glial cells, astrocytes are more efficient at DNA repair, as studies have shown that mitochondrial DNA oxidative damage [212] and O^6^-methylguanine [213] are more efficiently repaired in astrocytes than oligodendrocytes or microglia. It remains unknown whether DNA repair is affected in ALS astrocytes. However, PARP expression has been suggested to be increased in sALS astrocytes compared to controls, which could indicate DDR dysfunction [130]. Additionally, overexpressing SIRT6, which is involved in DDR and DNA repair, in primary astrocytes from *SOD1*-ALS mice reduces their toxicity to motor neurons [214].

## Conclusion

DNA damage is a common feature of sALS and fALS motor neurons (Table 4), and has been implicated in Western Pacific ALS, strongly suggesting it is involved in motor neuron degeneration. It remains unproven whether DNA damage is a direct cause of motor neuron degeneration in ALS or whether it is a consequence of other disease mechanisms. While several genes associated with fALS are thought to play a role in the DDR, they also have several other functions within the cell which may have a greater contribution to motor neuron degeneration. It is also worth noting the current limitations of the field. Most of the studies examining mechanisms of DNA damage in ALS have used 2D cell culture models, which are inherently limited at capturing the complexity of in vivo systems, and the field would benefit from using more relevant models, such as 3D organoid cultures [215]. In addition, many of the studies described in this review have used low-resolution methods to study DNA damage in ALS, and none so far have attempted to profile DNA damage across the genome, as has been done recently in neurons [216].

**Table 4:** Summary of studies of DNA damage in ALS

Type of ALS	Model	Tissue/cell type	Controls	DNA damage assay	Method	Reference
*C9ORF72*-ALS	Patient iPSCs	hiPSC-derived motor neurons	Age-matched healthy controls	Comet assay, γH2AX	ICC	[56]
*C9ORF72*-ALS	Post-mortem	Spinal cord	Sex-matched non-ALS controls	γH2AX	IHC	[59]
*C9ORF72*-ALS	Post-mortem, cell model	Lumbar spinal cord, SH-SY5Y human neuroblastoma cells transfected with DRPs	Age-matched healthy control tissue, cells expressing empty vector	γH2AX, 53BP1	ICC, IHC, immunoblot	[58]
*C9ORF72*-ALS	Patient iPSCs	hiPSC-derived motor neurons	Unaffected controls	γH2AX	Western blotting	[60]
*C9ORF72*-ALS, *NEK1*-ALS	Patient iPSCs	hiPSCs and hiPSC-derived motor neurons	Unspecified control cell lines	Comet assay, γH2AX	ICC	[57]
*FUS*-ALS	Mouse model	Cortex and spinal cord	Non-transgenic mice	Comet assay, γH2AX	IHC, Western blotting	[108]
*FUS*-ALS	Post-mortem	Motor cortex	NND controls	γH2AX	IHC	[105]
*FUS*-ALS	Post-mortem, patient iPSCs	Lumbar spinal cord, hiPSC-motor neurons	Age-matched healthy control tissue and cells and isogenic control cells	γH2AX	ICC, IHC	[106]
*FUS*-ALS	Patient iPSCs	hiPSC-motor neurons	Healthy controls and isogenic controls	Comet assay, γH2AX	ICC	[107]
sALS	Patient tissue	Blood serum	Healthy age and sex-matched controls	OdG	ELISA	[137]
***s*ALS**	**Patient tissue**	**Bone marrow MSCs**	**Healthy controls**	**γH2AX**	**ICC**	[133]
sALS	Patient tissue	CSF	Healthy age-matched controls	OdG	HPLC	[135]
sALS	Patient tissue	Urine	Healthy relatives as controls	OdG	ELISA, HPLC	[136]
***s*ALS**	**Patient tissue**	**Whole blood**	**Age-matched controls**	**Comet assay**	**N/A**	[132]
sALS	Patient tissue	Dermic fibroblasts	Healthy age and sex-matched controls	γH2AX	ICC	[134]
sALS, fALS	Patient tissue	CSF	Healthy controls	OdG	ELISA	[82]
sALS, fALS	Patient tissue	Urine, CSF and blood plasma	OND and healthy controls	OdG	LCEC	[81]
sALS, fALS	Post-mortem	Whole spinal cord, motor cortex	OND and healthy controls	OdG	HPLC, IHC	[13]
**sALS, fALS**	**Post-mortem**	**Motor cortex, frontal cortex**	**Mild cognitive impairment and healthy controls**	**γH2AX, OdG**	**IHC**	[150]
sALS, fALS	Post-mortem	Motor cortex, spinal cord	OND and age-matched controls	AP sites, OdG	AP assay, IHC	[103]
*SOD1*-ALS	Cell model	Human neuroglioma cells transfected with mutant SOD1	Untransfected and wild-type SOD1 transfected cells	Comet assay	N/A	[89]
*SOD1*-ALS	Cell model	SH-SY5Y human neuroblastoma cells transfected with mutant SOD1	Untransfected and wild-type SOD1 transfected cells	Comet assay, OdG	HPLC	[91]
*SOD1*-ALS	Cell model	Immortalised mouse motor neuron line NSC34 transfected with mutant SOD1	Untransfected and wild-type SOD1 transfected cells	Comet assay	N/A	[90]
*SOD1*-ALS	Cell model	NSC34 cells transfected with mutant SOD1	Untransfected and wild-type SOD1 transfected cells	OdG	ELISA	[92]
*SOD1*-ALS	Cell model	NSC34 cells stably expressing mutant SOD1	NSC34 cells stably expressing wild type hSOD1	γH2AX	ICC	[93]
***SOD1*-ALS**	**Mouse model**	**Cervical and thoracic spinal cord, primary motor neurons and astrocytes**	**Wild type mice**	**Comet assay, 53BP1, γH2AX**	**IHC, ICC**	[83]
*SOD1*-ALS	Mouse model	Lumbar spinal cord	Age-matched wild type mice	OdG	IHC	[85]
*SOD1*-ALS	Mouse model	Spinal cord, cortex and striatum	Age-matched mice	OdG	HPLC	[86]
*SOD1*-ALS	Mouse model	Whole spinal cord	Age-matched wild type mice	OdG	HPLC	[84]
*SOD1*-ALS	Mouse model	Cervical and lumbar spinal cord	hSOD1^G93A^-negative mice	γH2AX	IHC	[97]
*SOD1*-ALS	Mouse model	Spinal cord	Wild type mice	γH2AX	IHC	[87]
***TARDBP*-ALS**	**Patient iPSCs**	**hiPSC-motor neurons**	**Healthy controls**	**γH2AX**	**ICC**	[116]
*TARDBP*-ALS	Patient tissue	Spinal cord extract	Age-matched controls	γH2AX	IHC, Western	[117]
Unspecified	Post-mortem	Cervical spinal cord	Age-matched controls	OdG	HPLC	[14]

Rows in Italic indicate papers where no increase in DNA damage was observed, remainder found increase in DNA damage

*OND *other neurological disease, *NND *non-neurological disease, *ICC *immunocytochemistry, *IHC *immunohistochemistry, *ELISA *enzyme-linked immunosorbent assay, *HPLC *high-performance liquid chromatography, *LCEC *liquid chromatography with electrochemical detection

**Fig. 5:**
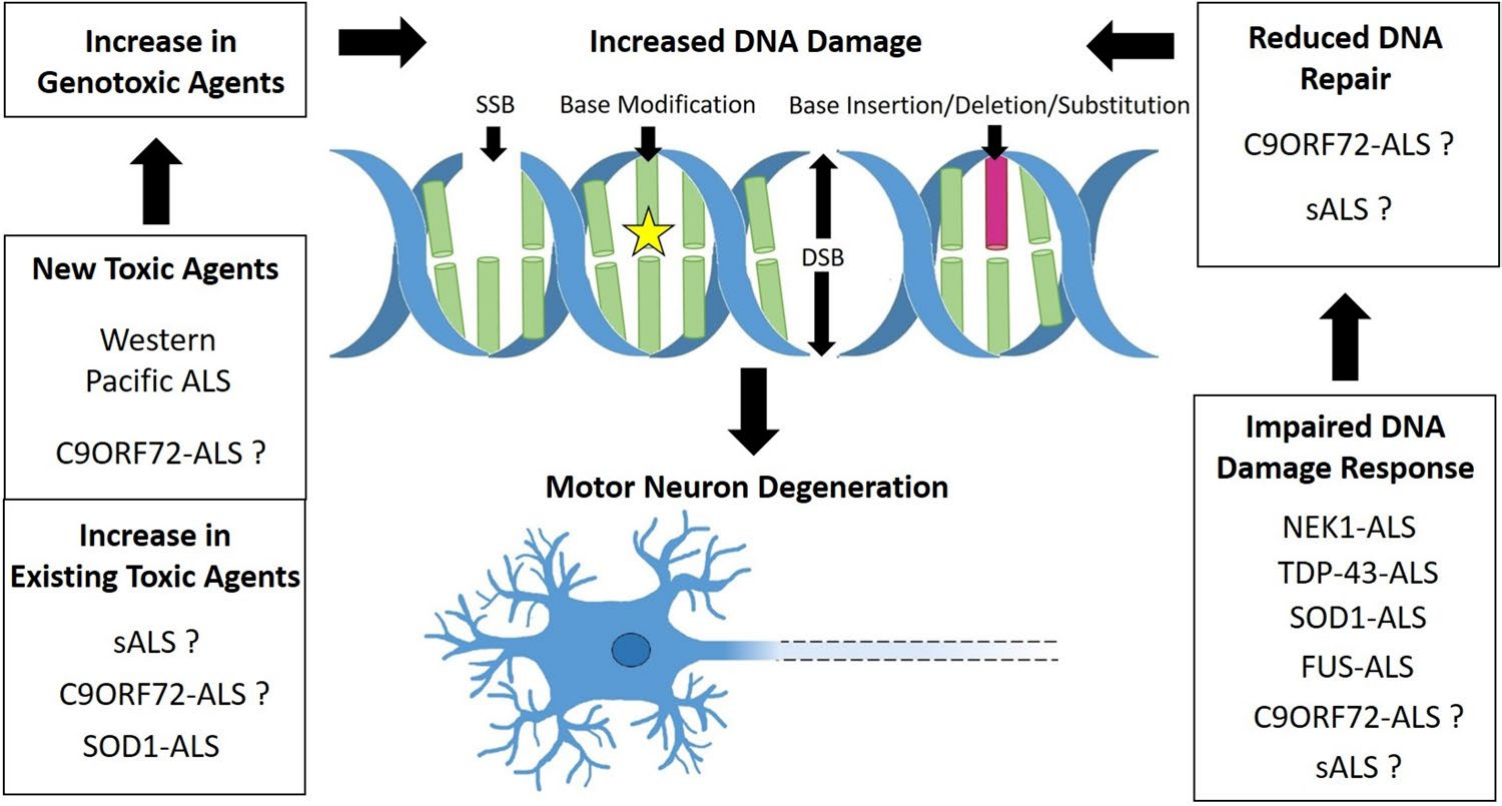
Proposed model for increased DNA damage in ALS. Increased DNA damage could arise through either an increase in exposure to genotoxic agents or a reduction in DNA repair mechanisms. An increase in genotoxic agents could occur through either exposure or generation of new toxic agents or an increase in existing agents. Reduced DNA repair could be due to defects in DNA repair pathways or because of inhibition or loss of components in the DNA damage response

Increased DNA damage in ALS cells could occur through two general mechanisms: DDR dysfunction or increased DNA damage agents. We propose that most forms of ALS are affected by at least one of these mechanisms (Fig. 5). DNA repair is thought to be less efficient in post-mitotic cells like motor neurons which, with other factors, could contribute to the vulnerability of motor neurons in ALS. Additionally, we discussed recent evidence indicating that ALS astrocytes may contribute to DNA damage in motor neurons and hasten motor neuron death through various mechanisms, including secretion of misfolded proteins and induction of autophagy dysregulation. Thus, boosting DNA repair or DDR pathways, or decreasing genotoxic agents could provide therapeutic benefit in ALS. In addition, evidence indicates that targeting ALS astrocytes with the aim to restore endogenous functions is a promising therapeutic strategy [217].

## Data Availability

Not applicable.
